# Comprehensive genomic analysis of five kindreds with multiple childhood leukemias: importance of individual functional analysis for rare *ETV6* germline variants

**DOI:** 10.1007/s13577-026-01422-z

**Published:** 2026-07-21

**Authors:** Ai Yamada, Shun Nagasawa, Midori Nakagawa, Sachiyo Kamimura, Nami Inoue, Hiroyoshi Watanabe, Masatoshi Takagi, Yuhki Koga, Dai Keino, Hideki Nakayama, Jun Yoshimura, Koichiro Doi, Jun Mitsui, Shoji Tsuji, Hiroshi Moritake

**Affiliations:** 1 Division of Pediatrics, Faculty of Medicine, University of Miyazaki, Miyazaki, Japan; 2 Department of Pediatrics, Graduate School of Biomedical Sciences, Tokushima University, Tokushima, Japan; 3 Department of Pediatrics and Developmental Biology, Institute of Science Tokyo, Tokyo, Japan; 4 Department of Pediatrics, Graduate School of Medical Sciences, Kyushu University, Fukuoka, Japan; 5 Department of Pediatrics, St. Marianna University School of Medicine, Kanagawa, Japan; 6 Department of Pediatrics, National Hospital Organization Kyushu Cancer Center, Fukuoka, Japan; 7 Graduate School of Frontier Sciences, The University of Tokyo, Tokyo, Japan; 8 School of Bioscience and Biotechnology, Tokyo University of Technology, Tokyo, Japan; 9 Department of Molecular Neurology, Graduate School of Medicine, The University of Tokyo, Tokyo, Japan

**Keywords:** Childhood leukemia, *ETV6* germline variant, Functional assay, Subcellular localization, Luciferase reporter assay

## Abstract

Germline variants in leukemia predisposition genes are increasingly detected in pediatric patients. Nevertheless, the rare variants identified in diagnostic samples may be misinterpreted without variant-level functional evaluation and cautious clinical interpretation. Whole-exome sequencing was performed on 28 individuals from five kindreds in which at least two children developed acute leukemia. Results identified a rare *ETV6* variant, c.604C > G (p.Arg202Gly), in monozygotic twins with *ETV6::RUNX1*-positive B-cell precursor acute lymphoblastic leukemia. To support variant interpretation, HeLa-cell populations stably expressing FLAG-tagged wild-type ETV6 and p.Arg202Gly and the known loss-of-function control p.Pro214Leu were established. Subcellular localization was assessed via immunofluorescence microscopy with quantitative scoring. Transcriptional repression was evaluated using luciferase reporter assays driven by *ETV6* target promoters (*MMP3* and *PF4*). p.Arg202Gly predominantly showed nuclear localization comparable to WT, whereas p.Pro214Leu was enriched in the cytoplasm. In the reporter assays, similar to WT, p.Arg202Gly retained repression activity. Meanwhile, p.Pro214Leu failed to repress both reporters. The in-silico prediction of nuclear export sequences suggested an additional export signal in p.Pro214Leu, but not in p.Arg202Gly. Collectively, these findings indicate that p.Arg202Gly behaves as WT-like in the assays performed and do not support a loss-of-function effect. Our study emphasizes the importance of variant-level functional assessment for rare *ETV6* variants to inform clinical interpretation and avoid overestimation of pathogenicity.

**Supplementary Information:**

The online version contains supplementary material available at 10.1007/s13577-026-01422-z.

## Introduction

Recent comprehensive genomic analyses have enabled the identification of germline variants as predisposition genes in pediatric cancer. Several germline predisposition genes, including *RUNX1*, *CEBPA*, *TP53*, *PAX5*, *IKZF1*, and *ETV6*, have been found to confer an inherited predisposition to leukemia [1]. The prevalence rate of germline variants among patients with leukemia is 4.4% [2]. Surveillance is important in patients with identified predisposing variants. Knowledge of such variants may improve the understanding of tumorigenesis, inform patient care, and enhance the genetic counseling of patients and families. The Kyushu Yamaguchi Children’s Cancer Study Group (KYCCSG) is a collaborative pediatric oncology network in the Kyushu–Yamaguchi region of Japan. Using the KYCCSG network, participating institutions were retrospectively surveyed to identify families in which more than one child developed acute leukemia. Whole-exome sequencing was performed on five such families. Consequently, we identified an extremely rare *ETV6* germline variant with unknown functional significance in monozygotic twins with *ETV6::RUNX1-*positive acute lymphoblastic leukemia (ALL) in one family.

*ETV6*, which is located at chromosome 12p13, encodes a transcriptional repressor in the ETS family with an essential role in hematopoiesis and embryonic development [3, 4]. *ETV6* comprises eight exons and contains three functional domains: a highly conserved N-terminal PNT domain that mediates homo- and heterodimerization; a central domain involved in repressive complex recruitment (SMRT, Sin3A, and NCOR) and nuclear export; and a C-terminal ETS DNA-binding domain [5]. *ETV6* is involved in the most common somatic translocation with *RUNX1* in up to 22% of childhood B-cell precursor (BCP) ALL [6]. Pre-leukemic clones carrying *ETV6::RUNX1* are frequently observed in neonatal cord blood. This finding provides evidence showing that the disease originates from a prenatal clone and requires secondary postnatal genetic aberrations [7–10].

In 2015, germline *ETV6* variants were first described as being responsible for familial thrombocytopenia with BCP ALL [11–13]. Subsequent studies have shown that several germline *ETV6* variants impair transcriptional repressor activity. However, a subset behaves similar to wild-type (WT-like), thereby underscoring the importance of variant-level functional evaluation if novel *ETV6* variants are identified [14].

Clinically, thrombocytopenia and occasional macrocytosis have been observed. Nevertheless, some carriers have normal platelet counts [12, 15–20].

In this study, whole-exome sequencing was performed on five kindreds with multiple childhood acute leukemias. A rare *ETV6* variant, c.604C > G (p.Arg202Gly), was identified in monozygotic twins with *ETV6::RUNX1*-positive BCP ALL. The functional consequences of rare *ETV6* variants vary significantly even within the same domain. Thus, variant-level functional characterization focusing on subcellular localization and transcriptional repression activity were conducted. This study aimed to provide experimental evidence supporting the clinical interpretation of this rare variant in the context of pediatric leukemia predisposition.

## Methods

### Patients and samples

The KYCCSG commenced clinical studies on hematological malignancies in 1984. From 1984 to 2012, 1276 patients (ALL, *n* = 955; acute myeloid leukemia, *n* = 281) were enrolled in clinical studies. Among these patients, four sibling pairs were included. To identify extremely rare germline variants with unknown functional significance, a comprehensive genomic analysis was conducted on five families, including three families from KYCCSG in which at least two children developed acute leukemia. Germline DNA was obtained from the peripheral blood or bone marrow samples collected during complete remission, defined as the absence of morphologically detectable leukemic blasts. These remission samples were used for germline variant calling to decrease contamination by tumor cells. Diagnostic (onset) samples were analyzed separately as a tumor material for comparison and the evaluation of somatic alterations. Written informed consent was obtained from the patients and/or their legal guardians, and the Institutional Review Board of the University of Miyazaki approved this study (approval number: 2013–071). Further, somatic identical mutations of leukemic cells with *ETV6::RUNX1* in twins were assessed to confirm whether preleukemic minor subclones arose prenatally.

### Exome sequencing

In total, 28 individuals from five families were subjected to exome sequencing, as described in a previous study [21]. Exonic sequences were captured using the Agilent SureSelect Human All Exon v5 + UTRs kit and sequenced on an Illumina HiSeq 2500 platform with 101-bp paired-end reads. Sequence reads were aligned to the human reference genome (hg19) using BWA (version 0.5.9-r16). Single-nucleotide variants (SNVs) and small insertions/deletions were identified using SAMtools (version 0.1.18) with default parameters. Variants were annotated based on RefSeq transcripts (September 2011). The known variants registered in public databases, including dbSNP build 135, the NHLBI Grand Opportunity Exome Sequencing Project, the 1000 Genomes Project, and other personal genome datasets, were filtered out. Non-synonymous SNVs and coding indels were extracted for further analysis.

### Filtering method

Supplementary Fig. 1 shows the SNV filtering strategy. Noncoding and synonymous variants and variants present in dbSNP build 135 were initially excluded. Variants not shared among affected individuals were then removed. The remaining variants were further evaluated to identify compound heterozygous, homozygous, or de novo variants. De novo variants were assessed as described in a previous study [22]. Variants shared by affected and unaffected individuals were excluded from the analysis. Similarly, variants in genes with a high level of evidence for inherited familial leukemia and genes implicated in carcinogenesis were extracted for further evaluation. Supplementary Table 1 presents these genes [1, 23–27].

### Cells

The HeLa cells were kindly provided by Professor Kazuhiro Morishita (Division of Tumor and Cellular Biochemistry, Department of Medical Sciences, Faculty of Medicine, University of Miyazaki, Miyazaki, Japan) and cultured in Dulbecco’s Modified Eagle Medium supplemented with 10% fetal bovine serum and 1% penicillin–streptomycin.

### Retroviral infection

Human *ETV6* sequences were amplified by polymerase chain reaction. The forward primer contained an EcoRI site, followed by a FLAG tag, and the reverse primer contained an XhoI site. The polymerase chain reaction products were digested with EcoRI and XhoI and then cloned into the EcoRI–XhoI-digested pMSCV-IRES-Puromycin vector [28]. ETV6 mutants (p.Arg202Gly and p.Pro214Leu) were generated using the KOD-Plus-Mutagenesis Kit (TOYOBO, Osaka, Japan). The p.Pro214Leu mutant, which is known to disturb ETV6 function [29], was used as a positive control. G3T-hi cells (Takara Bio, Shiga, Japan) were transiently transfected with pMSCV vectors and the pCL-Eco retrovirus packaging vector using polyethylenimine. HeLa cells were spin-infected (490 g for 45 min at 20 °C) with retroviral supernatant supplemented with polybrene (8 μg/mL).

### Immunofluorescence staining

To perform the functional analysis of the rare ETV6 variant, the localization of ETV6 was examined using established HeLa cells with a stable expression of FLAG-tagged WT, mutant ETV6 p.Arg202Gly, and p.Pro214Leu, separately. HeLa cells transfected with WT and mutated ETV6 (p.Arg202Gly and p.Pro214Leu) were fixed and permeabilized with 4% paraformaldehyde for 15 min at room temperature, washed in phosphate-buffered saline, and incubated in blocking buffer (5% normal goat serum and 0.3% Triton X/phosphate-buffered saline) for 60 min. After aspiration of the buffer, the cells were stained with anti-FLAG antibody (1:1000; Sigma) and then incubated with anti-mouse secondary antibody (1:2000; Sigma) for 2 h. The cells were stained with NucBlue™ Live ReadyProbes™ Reagent (Life Technologies, Grand Island, NY) for 20 min before imaging. The proportion of cells exhibiting FLAG signal predominantly in the nucleus or cytoplasm of WT and mutant *ETV6* was examined. Five individual experiments, with a total of at least 300 cells counted for each condition, were performed. One-way analysis of variance, followed by Dunnett’s multiple comparisons, was conducted to compare each variant with WT (*p < 0.05).

### Luciferase assay

To investigate the transcriptional regulatory properties, the repressive activity of the *ETV6* mutant compared with that of WT was analyzed using the luciferase reporter assay. Each promoter fragment of human *matrix metalloproteinase-3* (*MMP3*) and *platelet factor 4* (*PF4*), which are target genes of *ETV6*, was cloned into the pGL3-Basic Vector (Promega, Madison, WI). HeLa cells expressing WT or *ETV6* mutant were co-transfected with the pGL3 reporter construct with *MMP3* or *PF4* and pRL *Renilla* luciferase vector (Promega). Firefly and *Renilla* luciferase activities were examined using the Dual-Luciferase® Reporter Assay System (Promega).

### Nuclear export sequences

The number of nuclear export sequences (NESs) of WT, mutant *ETV6* p.Arg202Gly, and p.Pro214Leu was investigated using LocNES, which is freely available at http://prodata.swmed.edu/LocNES [30]. The query protein sequence in the FASTA format is the only required input.

## Results

### Whole-exome sequencing

**Fig. 1:**
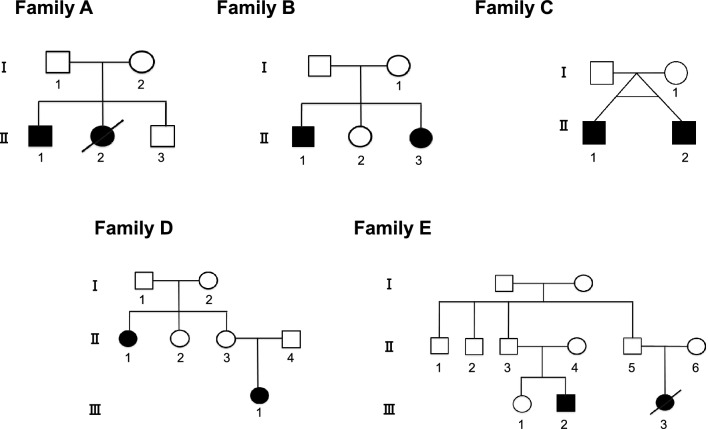
Pedigrees of families with acute leukemia who were enrolled in this study. The black-filled symbols represent patients with acute leukemia

**Fig. 2:**
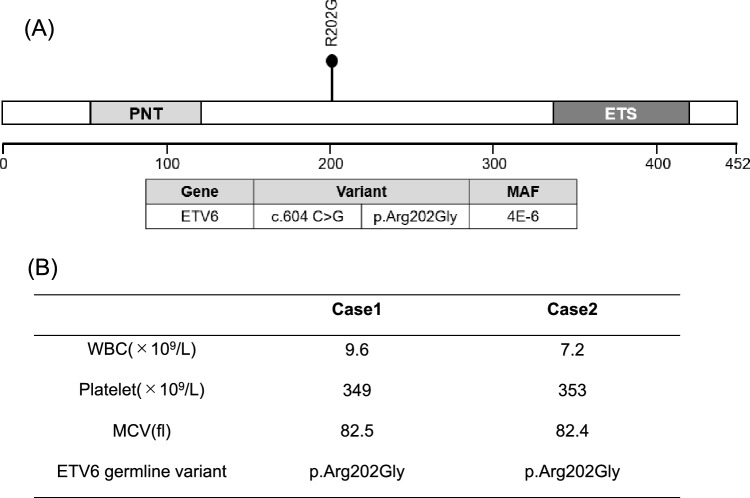
**A** Schematic representation of the ETV6 protein showing functional domains and the position of the variant identified in the twins. **B** Hematological data on complete remission in the twins harboring a germline *ETV6* variant in family C. *PNT* pointed N-terminal domain, *ETS* C-terminal DNA-binding domain, *MAF* minor allele frequency, *WBC* white blood cell, *MCV* mean corpuscular volume

Whole-exome sequencing was performed on 28 individuals from five kindreds with multiple cases of childhood acute leukemia (Fig. 1). The family history of patients who were enrolled in this study included second- to fourth-degree relatives who developed leukemia. Table 1 shows the clinical characteristics of each patient with leukemia. To evaluate shared germline candidates and exclude potential clonal hematopoiesis-associated findings, variants were examined using paired remission (germline) and diagnostic (tumor) samples obtained from affected individuals. De novo mutations shared among the affected individuals were not detected in each family. However, owing to the unavailability of samples from the patients’ fathers, de novo mutations could not be evaluated for families B and C. We subsequently searched for variants at the locus within the genes linked with hereditary leukemia or cancer predisposition. In family C, we identified a heterozygous *ETV6* variant, c.604 C > G (p.Arg202Gly), in the affected monozygotic twins (Fig. 2A). This variant was not registered in the databases of dbSNP build 135 and the Human Genetic Variation at the time of analysis in 2013–2014. However, it is now assigned with an SNP number (rs567885519). This variant is extremely rare in gnomAD (allele frequency: 0.00000186).

**Table 1:** Clinical characteristics of patients with familial leukemia

Family	Types of leukemia	Age at diagnosis (years)	Karyotype	Chimeric transcript
A				
Ⅱ-1	BCP-ALL	7	t(11;19)(q23;p13)	*KMT2A::MLLT1*
Ⅱ-2	Infant ALL	0	t(4;11)(q21;q23)	*KMT2A::AFF1*
B				
Ⅱ-1	BCP-ALL	11	46,XY	None
Ⅱ-3	BCP-ALL	6	46,XX	None
C				
Ⅱ-1	BCP-ALL	3	t(12;21)(p13;q22)	*ETV6::RUNX1*
Ⅱ-2	BCP-ALL	4	t(12;21)(p13;q22)	*ETV6::RUNX1*
D				
Ⅱ-1	BCP-ALL	8	46,XX	None
Ⅲ-1	BCP-ALL	0	45, XX, -6, der(7)t(6;7)(p11;q11.2), der(9)t(7;9)(q11.2;p13), add(15)(q24)	None
E				
Ⅲ-2	AML	12	t(16;21)(p11.2;q22)	*FUS::ERG*
Ⅲ-3	AML	2	45,XX,10,der(11)t(10;11)(q11.2;q23), del(12)(p11.2)	*KMT2A::MLLT10*

*ALL* acute lymphoblastic leukemia, *BCP-ALL* B-cell precursor acute lymphoblastic leukemia, *AML* acute myeloid leukemia

A male proband (case 1) and his twin (case 2) were diagnosed at 3 and 4 years of age, respectively, with *ETV6::RUNX1*-positive BCP ALL. Both patients were treated according to the Japanese Children’s Cancer and Leukemia Study Group standard-risk ALL protocol (UMIN000010488). They have been in complete remission for 12 and 11 years, respectively. The platelet counts and the mean corpuscular volume of red blood cells of these patients during complete remission were within the normal ranges (Fig. 2B).

### Immunofluorescence staining

**Fig. 3:**
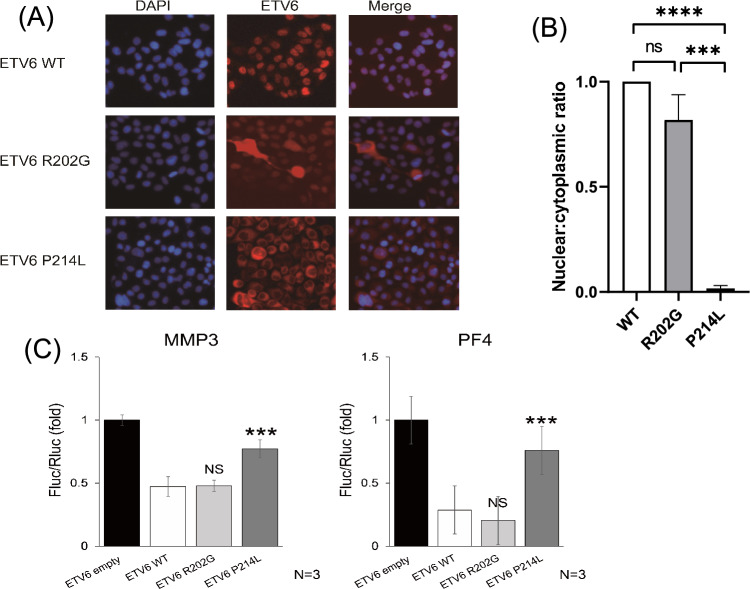
**A** Fluorescence images of HeLa cells expressing FLAG-tagged wild-type (WT), p.Arg202Gly, or p.Pro214Leu ETV6. **B** Quantification of nuclear-to-cytoplasmic fluorescence intensity ratios from five independent experiments. The bars represent mean ± standard deviation. Statistical analysis was performed using one-way analysis of variance, followed by Dunnett’s multiple comparisons comparing each variant with WT (***p < 0.0005, ****p < 0.0001). *WT* wild-type, *n.s.* not significant. **C** Effects of transfection-mediated repression of mutant ETV6. The firefly to *Renilla* luminescence ratios (Fluc/Rluc) were calculated to control for transfection efficiency. The promoter activities of *MMP3* and *PF4* were compared. The bars show the mean fold change in the Fluc/Rluc ratio relative to the empty vector. The data indicate three independent experiments. Unpaired *t*-tests were performed to compare each condition to the wild-type (***p < 0.005). *WT* wild-type, *n.s.* not significant, *MMP3* matrix metalloproteinase-3, *PF4* platelet factor 4

Fluorescence microscopy showed that WT ETV6 and p.Pro214Leu were predominantly located in the nucleus and cytoplasm, respectively (Fig. 3A). Similar to WT, p.Arg202Gly also predominantly showed nuclear localization, with a faint cytoplasmic signal observed in a subset of cells. Quantitative scoring revealed a significant cytoplasmic shift for p.Pro214Leu compared with WT. However, p.Arg202Gly did not differ significantly from WT (Fig. 3B).

### Luciferase assay

In luciferase reporter assays, WT *ETV6* repressed both *MMP3* and *PF4* promoter activities compared with the empty vector. Meanwhile, p.Pro214Leu did not repress either reporter (Fig. 3C). Similar to WT, p.Arg202Gly retained repression activity across both reporters, thereby supporting a WT-like transcriptional repression profile in this assay system (Fig. 3C).

### Nuclear export sequences as predicted by LocNES

LocNES analysis predicted one additional NES in p.Pro214Leu *ETV6* compared with WT *ETV6*. In contrast, the amino acid substitution (from Arg to Gly) at codon 202 did not alter the predicted number of NESs (Supplementary Table 2). Further, previously reported WT-like missense variants located in the central domain did not increase, as reported in a previous study [14].

These in silico predictions are consistent with the cytoplasmic enrichment observed for p.Pro214Leu. However, experimental validation of nuclear export activity was beyond the scope of this study.

## Discussion

This study analyzed five kindreds with multiple cases of childhood acute leukemia and identified a rare ETV6 variant, p.Arg202Gly, in monozygotic twins with *ETV6::RUNX1*-positive BCP ALL. The germline *ETV6* variants exhibit heterogeneous functional effects and various clinical penetrance. Therefore, variant-level evaluation is essential, particularly if rare variants are detected during genetic testing in pediatric patients with leukemia and their relatives.

Pathogenic *ETV6* variants have been found to cluster predominantly within the DNA-binding domain, which presumably leads to a decrease in transcription repressor activity and may have dominant negative effects [14]. In contrast, only a limited number of missense variants have been described in the central domain, including p.Pro214Leu, which has been functionally characterized as a loss-of-function variant [29]. In terms of clinical manifestation, patients harboring impaired *ETV6* variants commonly exhibit mild-to-moderate thrombocytopenia and an increased risk of leukemia [5, 12, 13, 15–20]. Children with ALL who presented with *ETV6* variants have been diagnosed at a relatively older age compared with sporadic cases (10.2 vs. 4.7 years) [11]. In our cases, the twins were diagnosed at 3 and 4 years of age and did not exhibit thrombocytopenia during complete remission, features that are not typical of classical loss-of-function *ETV6* variants.

Functionally, the p.Arg202Gly *ETV6* variant was evaluated using subcellular localization and transcriptional repression assays. In contrast to p.Pro214Leu, p.Arg202Gly predominantly displayed nuclear localization comparable to WT *ETV6* and retained repression of target promoters. Collectively, these findings indicate that p.Arg202Gly behaves in a WT-like manner and does not support a loss-of-function effect in the assays performed. The difference in subcellular localization between p.Pro214Leu and p.Arg202Gly, both located within the central domain of *ETV6*, may be attributed to differences in predicted NESs. Consistent with this interpretation, previously reported WT-like missense variants in the central domain do not introduce additional export signals, indicating that the leucine substitution in p.Pro214Leu might contribute to the formation of an additional nuclear export sequence. However, we cannot completely exclude the possibility that p.Arg202Gly contributes to leukemogenesis through mechanisms not assessed in this study (e.g., genomic stability, hematopoietic stem cell function, or clonal evolution). Especially, ETV6 is implicated in hematopoietic stem cell maintenance and lineage commitment [31]. Moreover, *ETV6::RUNX1*-positive ALL is widely known to arise through a multistep leukemogenic process, wherein a prenatal *ETV6::RUNX1* fusion establishes a pre-leukemic clone that subsequently acquires secondary genetic alterations [32]. Therefore, it remains possible that the p.Arg202Gly germline variant exerts subtle effects on hematopoietic development or pre-leukemic clonal evolution in these twins who have pre-leukemic clones with prenatal *ETV6::RUNX1* fusion.

In this study, five kindreds with multiple childhood leukemias were analyzed. However, a pathogenic germline variant was not identified in four families after filtering for known leukemia predisposition genes. Several affected individuals in these families harbored leukemia-defining somatic drivers, suggesting that familial clustering may reflect shared environmental or stochastic factors, low-penetrance susceptibility alleles, or undiscovered predisposition mechanisms rather than a single high-penetrance germline mutation. These findings emphasize the complexity of interpreting familial leukemia aggregation. Further, they highlight that rare variants detected via sequencing require cautious functional and clinical evaluation before being considered causative.

This study has several limitations. First, the number of affected individuals in each family was small, thereby limiting the statistical power to detect low-penetrance predisposition alleles. Second, de novo variants could not be assessed in families without paternal samples. Third, because our analysis primarily focused on identifying shared germline variants among affected individuals, somatic variants and copy number alterations were not comprehensively evaluated. Fourth, the functional assays were based on overexpression systems using HeLa cells and did not address the other aspects of *ETV6* biology, including DNA-binding capacity, protein–protein interactions, and hematopoietic context-specific effects. Although HeLa cells are widely used for evaluating ETV6 localization and transcriptional repression [12, 13], functional analyses using hematopoietic cell models may provide additional insights into the biological significance of ETV6 variants. Finally, although children who were not affected at enrollment did not develop leukemia after > 10 years of follow-up, future disease development cannot be completely excluded.

In conclusion, this study identified a rare *ETV6* germline variant, p.Arg202Gly, in monozygotic twins with *ETV6::RUNX1*-positive BCP ALL via a comprehensive genomic analysis. Functional analyses showed no evidence of impaired *ETV6* activity in the assays performed. Therefore, this variant should be interpreted cautiously with respect to leukemia predisposition rather than being assumed pathogenic. Our findings emphasize the importance of variant-level functional evaluation of newly identified variants for guiding appropriate genetic counseling and surveillance decisions while preventing overestimation of pathogenicity.

## Supplementary Information

Below is the link to the electronic supplementary material.

Supplementary file1 (DOCX 661 KB) Whole-exome sequencing and familial genomic filtering strategy used to identify candidate germline variants

Supplementary file2 (DOCX 22 KB)

Supplementary file3 (DOCX 23 KB)

## Data Availability

The datasets generated and/or analyzed during the current study are not publicly available due to ethical restrictions and consent limitations. However, they are available from the corresponding author upon reasonable request.
