# Supplementary material for: Comprehensive genomic analysis of five kindreds with multiple childhood leukemias: importance of individual functional analysis for rare ETV6 germline variants
Source: Hum Cell. 2026 Jul 21;39(8):113. doi: 10.1007/s13577-026-01422-z (PMC13388546; doi:10.1007/s13577-026-01422-z)
Supplement: Supplementary file 1 — Supplementary file1 (DOCX 661 KB) Whole-exome sequencing and familial genomic filtering strategy used to identify candidate germline variants [file 13577_2026_1422_MOESM1_ESM.docx]

**Supplementary Figure 1**

**
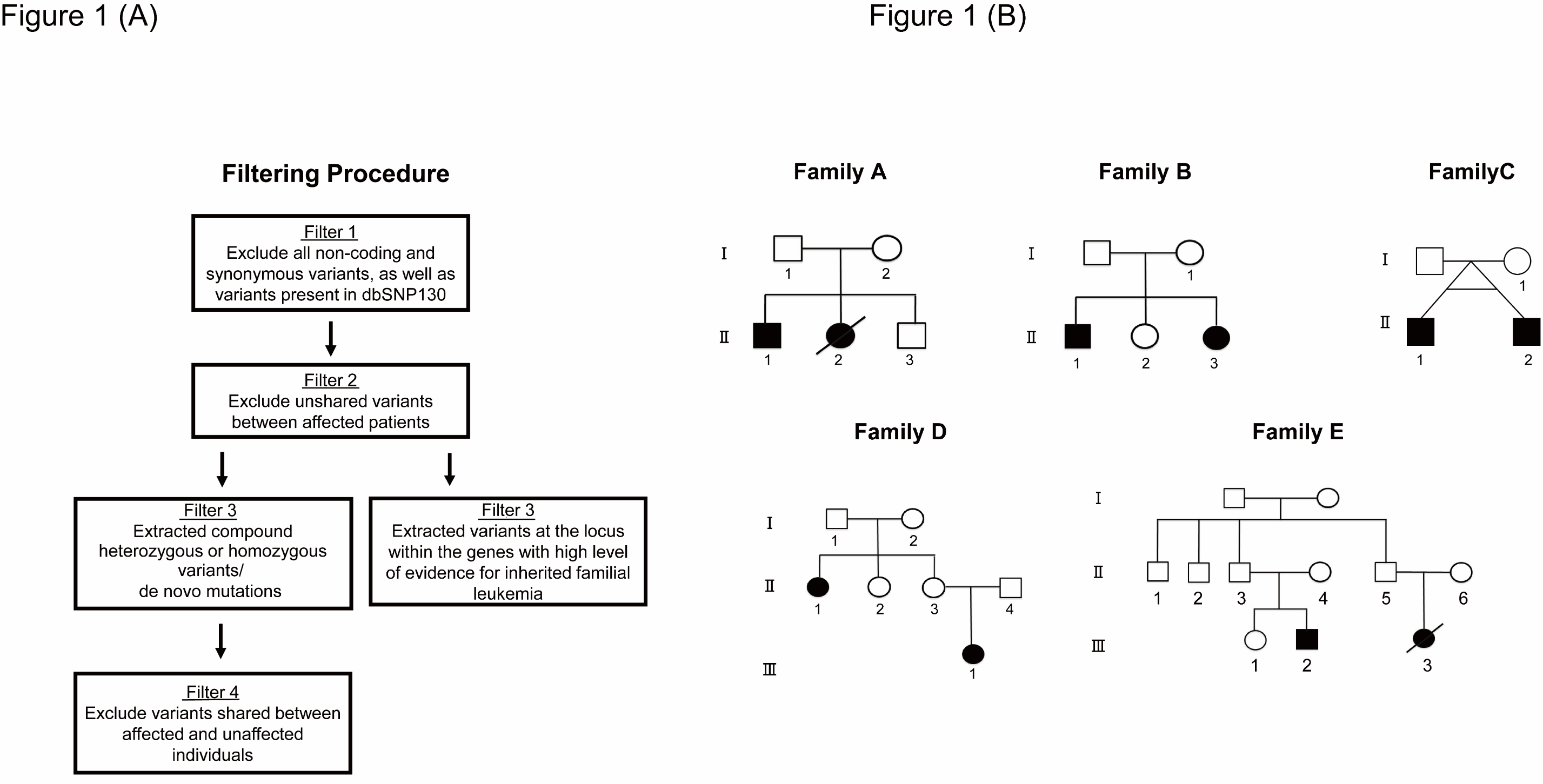
**

Filter 1

Exclude all non-coding and synonymous variants, as well as variants present in dbSNP build 135
