# Supplementary material for: Comprehensive genomic analysis of five kindreds with multiple childhood leukemias: importance of individual functional analysis for rare ETV6 germline variants
Source: Hum Cell. 2026 Jul 21;39(8):113. doi: 10.1007/s13577-026-01422-z (PMC13388546; doi:10.1007/s13577-026-01422-z)
Supplement: Supplementary file 2 — Supplementary file2 (DOCX 22 KB) [file 13577_2026_1422_MOESM2_ESM.docx]

**Supplementary Table 1. Genes with evidence for inherited predisposition to familial leukemia**

|  | AML | ALL | References |
| --- | --- | --- | --- |
| High penetrance susceptibility | RUNX1, CEBPA, GATA2, DDX41, ANKRD26, SRP72, TERC, TERT, SAMD9, SAMD9L | ETV6, IKZF1, PAX5 | 1), 18) - 21) |
| Tumor suppressor gene syndromes | TP53, NF1, PTPN11, CBL | | 21) |
| DNA repair gene syndromes | PMS2, MSH6, MLH1, MSH2, FANC A-E, BRCA, ATM, NBS1, BLM, WRN, RECQL4 | | 21) |

AML: acute myeloid leukemia; ALL: acute lymphoblastic leukemia

**Genes involved in hereditary cancer syndrome or somatic mutations in cancer**

| ABL1 | ACD | ACTN4 | AKT1 | AKT2 | AKT3 | ALK | APC |
| --- | --- | --- | --- | --- | --- | --- | --- |
| ARAF | ARID1A | ARID2 | ATM | AXIN1 | AXL | B2M | BAP1 |
| BARD1 | BCL2L11/BIM | BLM | BRAF | BRCA1 | BRCA2 | CCND1 | CCNE1 |
| CD274/PD-L1 | CDH1 | CDK12 | CDK4 | CDK6 | CDKN2A | CEBPA | CHEK2 |
| CREBBP | CRKL | CTNNA1 | CTNNB1 | CUL3 | DDR2 | DDX41 | DICER1 |
| EGFR | ENO1 | EP300 | EPCAM | ERBB2/HER2 | ERBB3 | ERBB4 | ESR1/ER |
| ETV6 | EZH2 | FANCM | FBXW7 | FGFR1 | FGFR2 | FGFR3 | FGFR4 |
| FH | FLCN | FLT3 | GATA2 | GNA11 | GNAQ | GNAS | GREM1 |
| HOXB13 | HRAS | IDH1 | IDH2 | IGF1R | IGF2 | IKZF1 | IL7R |
| JAK1 | JAK2 | JAK3 | KDM6A/UTX | KEAPI | KIT | KLHDC8B | KRAS |
| MAP2K1/MEK1 | MAP2K2/MEK2 | MAP2K4 | MAP3K1 | MAP3K4 | MDM2 | MDM4 | MEN1 |
| MET | MLH1 | MSH2 | MSH2/6 | MSH3 | MSH6 | MTAP | MTOR |
| MUTYH | MYC | MYCN | NBN | NF1 | NF2 | NFE2L2/Nerf2 | NOTCH1 |
| NOTCH2 | NOTCH3 | NPAT | NRAS | NRG1 | NTHL1 | NTRK1 | NTRK2 |
| NTRK3 | NTSC2 | PALB2 | PAX5 | PBRM1 | PDGFRA | PDGFRB | PIK3CA |
| PIK3R1 | PIK3R2 | PMS2 | POLD1 | POLE | POT1 | PRKCI | PTCH1 |
| PTEN | RABL3 | RAC1 | RAC2 | RAD51C | RAD51D | RAF1/CRAF | RB1 |
| RECQL | RET | RHOA | RNF43 | ROS1 | RPS20 | RUNX1 | SAMD9 |
| SAMD9L | SETBP1 | SETD2 | SMAD4 | SMARCA4 | SMARCA4/BRG1 | SMARCB1 | SMO |
| STAT3 | STK11/LKB1 | T790M | TERF2IP | TERT | TP53 | TSC1 | TSC2 |
| VEGFR2 | VHL | WDR77 | WT1 |  |  |  |  |
