# Supplementary material for: Comprehensive genomic analysis of five kindreds with multiple childhood leukemias: importance of individual functional analysis for rare ETV6 germline variants
Source: Hum Cell. 2026 Jul 21;39(8):113. doi: 10.1007/s13577-026-01422-z (PMC13388546; doi:10.1007/s13577-026-01422-z)
Supplement: Supplementary file 3 — Supplementary file3 (DOCX 23 KB) [file 13577_2026_1422_MOESM3_ESM.docx]

**Supplementary Table 2. Nuclear export sequences of ETV6, as predicted by LocNES**

| WT ETV6 |  |  |  |
| --- | --- | --- | --- |
| **Protein Name** | **Position** | **Sequence** | **Score** |
| >LocNES1550139579_0 | 80-94 | SSTPLHVPVPRALRM | 0.06 |
| >LocNES1550139579_0 | 116-130 | DVAQWLKWAENEFSL | 0.02 |
| >LocNES1550139579_0 | 316-330 | VIQLMPSPIMHPLIL | 0.438 |
| >LocNES1550139579_0 | 357-371 | LSHREDLAYMNHIMV | 0.031 |
| >LocNES1550139579_0 | 359-373 | HREDLAYMNHIMVSV | 0.089 |
| ETV6 R202G |  |  |  |
| **rotein Name** | **Position** | **Sequence** | **Score** |
| >LocNES241877553_0 | 80-94 | SSTPLHVPVPRALRM | 0.06 |
| >LocNES241877553_0 | 116-130 | DVAQWLKWAENEFSL | 0.02 |
| >LocNES241877553_0 | 316-330 | VIQLMPSPIMHPLIL | 0.438 |
| >LocNES241877553_0 | 357-371 | LSHREDLAYMNHIMV | 0.031 |
| >LocNES241877553_0 | 359-373 | HREDLAYMNHIMVSV | 0.089 |
| ETV6 P214L |  |  |  |
| **Protein Name** | **Position** | **Sequence** | **Score** |
| >LocNES489367305_0 | 80-94 | SSTPLHVPVPRALRM | 0.053 |
| >LocNES489367305_0 | 116-130 | DVAQWLKWAENEFSL | 0.019 |
| >LocNES489367305_0 | 251-265 | PLRSPLDNMIRRLSL | 0.611 |
| >LocNES489367305_0 | 316-330 | VIQLMPSPIMHPLIL | 0.24 |
| >LocNES489367305_0 | 357-371 | LSHREDLAYMNHIMV | 0.031 |
| >LocNES489367305_0 | 359-373 | HREDLAYMNHIMVSV | 0.089 |
| ETV6 R127Q |  |  |  |
| **Protein Name** | **Position** | **Sequence** | **Score** |
| >LocNES412690009_0 | 80-94 | SSTPLHVPVPRALRM | 0.06 |
| >LocNES412690009_0 | 116-130 | DVAQWLKWAENEFSL | 0.02 |
| >LocNES412690009_0 | 316-330 | VIQLMPSPIMHPLIL | 0.438 |
| >LocNES412690009_0 | 357-371 | LSHREDLAYMNHIMV | 0.031 |
| >LocNES412690009_0 | 359-373 | HREDLAYMNHIMVSV | 0.089 |
| ETV6 V166M |  |  |  |
| **Protein Name** | **Position** | **Sequence** | **Score** |
| >LocNES1933415715_0 | 80-94 | SSTPLHVPVPRALRM | 0.06 |
| >LocNES1933415715_0 | 116-130 | DVAQWLKWAENEFSL | 0.02 |
| >LocNES1933415715_0 | 316-330 | VIQLMPSPIMHPLIL | 0.438 |
| >LocNES1933415715_0 | 357-371 | LSHREDLAYMNHIMV | 0.031 |
| >LocNES1933415715_0 | 359-373 | HREDLAYMNHIMVSV | 0.089 |
| ETV6 R181H |  |  |  |
| **Protein Name** | **Position** | **Sequence** | **Score** |
| >LocNES1745098404_0 | 80-94 | SSTPLHVPVPRALRM | 0.06 |
| >LocNES1745098404_0 | 116-130 | DVAQWLKWAENEFSL | 0.02 |
| >LocNES1745098404_0 | 316-330 | VIQLMPSPIMHPLIL | 0.438 |
| >LocNES1745098404_0 | 357-371 | LSHREDLAYMNHIMV | 0.031 |
| >LocNES1745098404_0 | 359-373 | HREDLAYMNHIMVSV | 0.089 |
| ETV6 R199Q |  |  |  |
| **Protein Name** | **Position** | **Sequence** | **Score** |
| >LocNES2000474660_0 | 80-94 | SSTPLHVPVPRALRM | 0.06 |
| >LocNES2000474660_0 | 116-130 | DVAQWLKWAENEFSL | 0.02 |
| >LocNES2000474660_0 | 316-330 | VIQLMPSPIMHPLIL | 0.438 |
| >LocNES2000474660_0 | 357-371 | LSHREDLAYMNHIMV | 0.031 |
| >LocNES2000474660_0 | 359-373 | HREDLAYMNHIMVSV | 0.089 |
| ETV6 L201P |  |  |  |
| **Protein Name** | **Position** | **Sequence** | **Score** |
| >LocNES952651499_0 | 80-94 | SSTPLHVPVPRALRM | 0.06 |
| >LocNES952651499_0 | 116-130 | DVAQWLKWAENEFSL | 0.02 |
| >LocNES952651499_0 | 316-330 | VIQLMPSPIMHPLIL | 0.438 |
| >LocNES952651499_0 | 357-371 | LSHREDLAYMNHIMV | 0.031 |
| >LocNES952651499_0 | 359-373 | HREDLAYMNHIMVSV | 0.089 |
| ETV6 R202Q |  |  |  |
| **Protein Name** | **Position** | **Sequence** | **Score** |
| >LocNES859213392_0 | 80-94 | SSTPLHVPVPRALRM | 0.06 |
| >LocNES859213392_0 | 116-130 | DVAQWLKWAENEFSL | 0.02 |
| >LocNES859213392_0 | 316-330 | VIQLMPSPIMHPLIL | 0.438 |
| >LocNES859213392_0 | 357-371 | LSHREDLAYMNHIMV | 0.031 |
| >LocNES859213392_0 | 359-373 | HREDLAYMNHIMVSV | 0.089 |
| ETV6 P223L |  |  |  |
| **Protein Name** | **Position** | **Sequence** | **Score** |
| >LocNES142148561_0 | 80-94 | SSTPLHVPVPRALRM | 0.06 |
| >LocNES142148561_0 | 116-130 | DVAQWLKWAENEFSL | 0.02 |
| >LocNES142148561_0 | 316-330 | VIQLMPSPIMHPLIL | 0.515 |
| >LocNES142148561_0 | 357-371 | LSHREDLAYMNHIMV | 0.031 |
| >LocNES142148561_0 | 359-373 | HREDLAYMNHIMVSV | 0.089 |
| ETV6 R259Q |  |  |  |
| **Protein Name** | **Position** | **Sequence** | **Score** |
| >LocNES1566165609_0 | 80-94 | SSTPLHVPVPRALRM | 0.06 |
| >LocNES1566165609_0 | 116-130 | DVAQWLKWAENEFSL | 0.02 |
| >LocNES1566165609_0 | 316-330 | VIQLMPSPIMHPLIL | 0.438 |
| >LocNES1566165609_0 | 357-371 | LSHREDLAYMNHIMV | 0.031 |
| >LocNES1566165609_0 | 359-373 | HREDLAYMNHIMVSV | 0.089 |
| ETV6 A329T |  |  |  |
| **Protein Name** | **Position** | **Sequence** | **Score** |
| >LocNES794773578_0 | 80-94 | SSTPLHVPVPRALRM | 0.06 |
| >LocNES794773578_0 | 116-130 | DVAQWLKWAENEFSL | 0.02 |
| >LocNES794773578_0 | 316-330 | VIQLMPSPIMHPLIL | 0.438 |
| >LocNES794773578_0 | 357-371 | LSHREDLAYMNHIMV | 0.031 |
| >LocNES794773578_0 | 359-373 | HREDLAYMNHIMVSV | 0.089 |
